# Integrative Transkingdom Analysis of the Gut Microbiome in Antibiotic Perturbation and Critical Illness

**DOI:** 10.1128/mSystems.01148-20

**Published:** 2021-03-16

**Authors:** Bastiaan W. Haak, Ricard Argelaguet, Cormac M. Kinsella, Robert F. J. Kullberg, Jacqueline M. Lankelma, Martin Deijs, Michelle Klein, Maarten F. Jebbink, Floor Hugenholtz, Sarantos Kostidis, Martin Giera, Theodorus B. M. Hakvoort, Wouter J. de Jonge, Marcus J. Schultz, Tom van Gool, Tom van der Poll, Willem M. de Vos, Lia M. van der Hoek, W. Joost Wiersinga

**Affiliations:** a Center for Experimental and Molecular Medicine, Amsterdam UMC, Location AMC, Amsterdam Infection and Immunity Institute, Amsterdam, The Netherlands; b European Molecular Biology Laboratory, European Bioinformatics Institute, Hinxton, Cambridge, United Kingdom; c Laboratory of Experimental Virology, Department of Medical Microbiology, Amsterdam UMC, Location AMC, Amsterdam, The Netherlands; d Center for Proteomics and Metabolomics, Leiden University Medical Center, Leiden, The Netherlands; e Tytgat Institute for Liver and Intestinal Research, Amsterdam UMC, Location AMC, Amsterdam, The Netherlands; f Department of Intensive Care, Amsterdam UMC, Location AMC, Amsterdam, The Netherlands; g Department of Parasitology, Amsterdam UMC, Location AMC, Amsterdam, The Netherlands; h Laboratory of Microbiology, Wageningen University, Wageningen, The Netherlands; i Research Programs Unit Immunobiology, Department of Bacteriology and Immunology, Helsinki University, Helsinki, Finland; j Department of Medicine, Division of Infectious Diseases, Amsterdam UMC, Location AMC, Amsterdam, The Netherlands; Mayo Clinic

**Keywords:** bacteriophages, fungi, multi-omics, data integration, bacteria, microbiome

## Abstract

Bacterial microbiota play a critical role in mediating local and systemic immunity, and shifts in these microbial communities have been linked to impaired outcomes in critical illness. Emerging data indicate that other intestinal organisms, including bacteriophages, viruses of eukaryotes, fungi, and protozoa, are closely interlinked with the bacterial microbiota and their host, yet their collective role during antibiotic perturbation and critical illness remains to be elucidated. We employed multi-omics factor analysis (MOFA) to systematically integrate the bacterial (16S rRNA), fungal (intergenic transcribed spacer 1 rRNA), and viral (virus discovery next-generation sequencing) components of the intestinal microbiota of 33 critically ill patients with and without sepsis and 13 healthy volunteers. In addition, we quantified the absolute abundances of bacteria and fungi using 16S and 18S rRNA PCRs and characterized the short-chain fatty acids (SCFAs) butyrate, acetate, and propionate using nuclear magnetic resonance spectroscopy. We observe that a loss of the anaerobic intestinal environment is directly correlated with an overgrowth of aerobic pathobionts and their corresponding bacteriophages as well as an absolute enrichment of opportunistic yeasts capable of causing invasive disease. We also observed a strong depletion of SCFAs in both disease states, which was associated with an increased absolute abundance of fungi with respect to bacteria. Therefore, these findings illustrate the complexity of transkingdom changes following disruption of the intestinal bacterial microbiome.

**IMPORTANCE** While numerous studies have characterized antibiotic-induced disruptions of the bacterial microbiome, few studies describe how these disruptions impact the composition of other kingdoms such as viruses, fungi, and protozoa. To address this knowledge gap, we employed MOFA to systematically integrate viral, fungal, and bacterial sequence data from critically ill patients (with and without sepsis) and healthy volunteers, both prior to and following exposure to broad-spectrum antibiotics. In doing so, we show that modulation of the bacterial component of the microbiome has implications extending beyond this kingdom alone, enabling the overgrowth of potentially invasive fungi and viruses. While numerous preclinical studies have described similar findings *in vitro*, we confirm these observations in humans using an integrative analytic approach. These findings underscore the potential value of multi-omics data integration tools in interrogating how different components of the microbiota contribute to disease states. In addition, our findings suggest that there is value in further studying potential adjunctive therapies using anaerobic bacteria or SCFAs to reduce fungal expansion after antibiotic exposure, which could ultimately lead to improved outcomes in the intensive care unit (ICU).

## INTRODUCTION

In recent years, widespread efforts have been dedicated to elucidating the impact of intestinal microorganisms in health and disease (1, 2). Animal studies have shown that broad-spectrum antibiotic modulation of the intestinal microbiota enhances susceptibility to enteric and systemic infections (3–5). In line with these preclinical findings, our group and others have observed that exposure to broad-spectrum antimicrobial therapy profoundly distorts the composition of the intestinal microbes of critically ill patients in the intensive care unit (ICU) (6–8). These disruptions within the intestinal environment enable the rapid expansion of opportunistic pathobionts and nosocomial infections, including infections with vancomycin-resistant enterococci as well as invasive disease by antibiotic-resistant *Enterobacteriaceae* (9–11).

Traditionally, viruses were considered solely pathogens; however, growing evidence suggests a more dynamic relationship between the virome and the host, mediated by direct interactions with the bacterial microbiome (12–15). Viruses influence immune development and shape tissue architecture (16, 17), and changes in the composition of viral communities have been associated with disease severity in inflammatory bowel disease (IBD), AIDS, and the development of graft-versus-host disease (GvHD) (12, 18, 19). Similarly, intestinal fungi have recently been acknowledged as a small but potentially important part of the intestinal ecosystem and have been shown to play a potentially immunomodulatory role in the development of colorectal cancer, IBD, and irritable bowel syndrome (IBS) (20–24).

While these findings provide clues that specific cross-kingdom interactions potentially contribute to or exacerbate disease, a large knowledge gap remains on the composition and interactions of fungi and viruses following exposure to broad-spectrum antibiotics, both in healthy volunteers and in patients with a critical illness. Moreover, there is a large gap between *in vitro* observations and confirmation of these patterns in humans. Hence, there is an increasing need for integrative computational frameworks that can systematically identify underlying patterns of variation across these communities in health and disease (12, 23).

## RESULTS AND DISCUSSION

### Experimental design.

To examine the extent of transkingdom associations during critical illness, we collected fecal samples from 33 patients (mean age, 62 years; 45% male) (see Table S1 in the supplemental material) admitted to the intensive care unit (ICU) of the Amsterdam University Medical Center, Location AMC. Of these patients, 24 were admitted with sepsis, while 9 patients had a noninfectious diagnosis (nonseptic ICU). All ICU patients were treated with between one and nine different classes of antimicrobial agents (Fig. S1). Thirteen healthy, nonsmoking, Caucasian male subjects (aged 18 to 25 years; mean age, 22 years) were evaluated as controls. Six healthy subjects received oral broad-spectrum antibiotics (ciprofloxacin at 500 mg every 12 h [q12h], vancomycin at 500 mg q8h, and metronidazole at 500 mg q8h) for 7 days, whereas 7 subjects did not receive antibiotics. Subjects were asked to collect fecal samples before antibiotic treatment and 1 day after completing the course of antibiotics.

10.1128/mSystems.01148-20.1 FIG S1 Overview of antibiotic exposure of the study cohort. Healthy volunteers are displayed at the top, septic patients are in the middle, and nonseptic ICU patients are displayed at the bottom. The black line indicates the length of stay (for sepsis and nonseptic ICU patients) or length of follow-up (for volunteers). Crosses indicate the days of fecal sample collection. Download FIG S1, TIF file, 0.8 MB. Copyright © 2021 Haak et al. 2021 Haak et al. https://creativecommons.org/licenses/by/4.0/ This content is distributed under the terms of the Creative Commons Attribution 4.0 International license.

10.1128/mSystems.01148-20.8 TABLE S1 Characteristics of all included patients admitted to the ICU with sepsis or nonseptic critical illness. Download Table S1, DOCX file, 0.02 MB. Copyright © 2021 Haak et al. 2021 Haak et al. https://creativecommons.org/licenses/by/4.0/ This content is distributed under the terms of the Creative Commons Attribution 4.0 International license.

We performed sequencing of the V3-V4 region of the bacterial 16S rRNA gene and the fungal intergenic transcribed spacer 1 (ITS1) rRNA gene, seeking to examine community compositions by characterizing fungal and bacterial sequences into exact amplicon sequencing variants (ASVs) (25). We simultaneously performed virus discovery cDNA-amplified fragment length polymorphism next-generation sequencing (VIDISCA-NGS) (26) using a validated virome-enriched library preparation (27, 28). Finally, the presence of Giardia lamblia, Cryptosporidium parvum, Entamoeba histolytica, Blastocystis hominis, and Dientamoeba fragilis was assessed by real-time PCR targeting the small-subunit (SSU) rRNA gene. Of note, the bacterial microbiomes of ICU patients (6) and volunteers (29) included in this study have been described previously by our group in two separate publications. For the purpose of this study, the bacterial microbiomes of patients and volunteers were resequenced to reduce batch effects.

### Composition and diversity of the bacterial, fungal, and viral microbiome.

**FIG 1:**
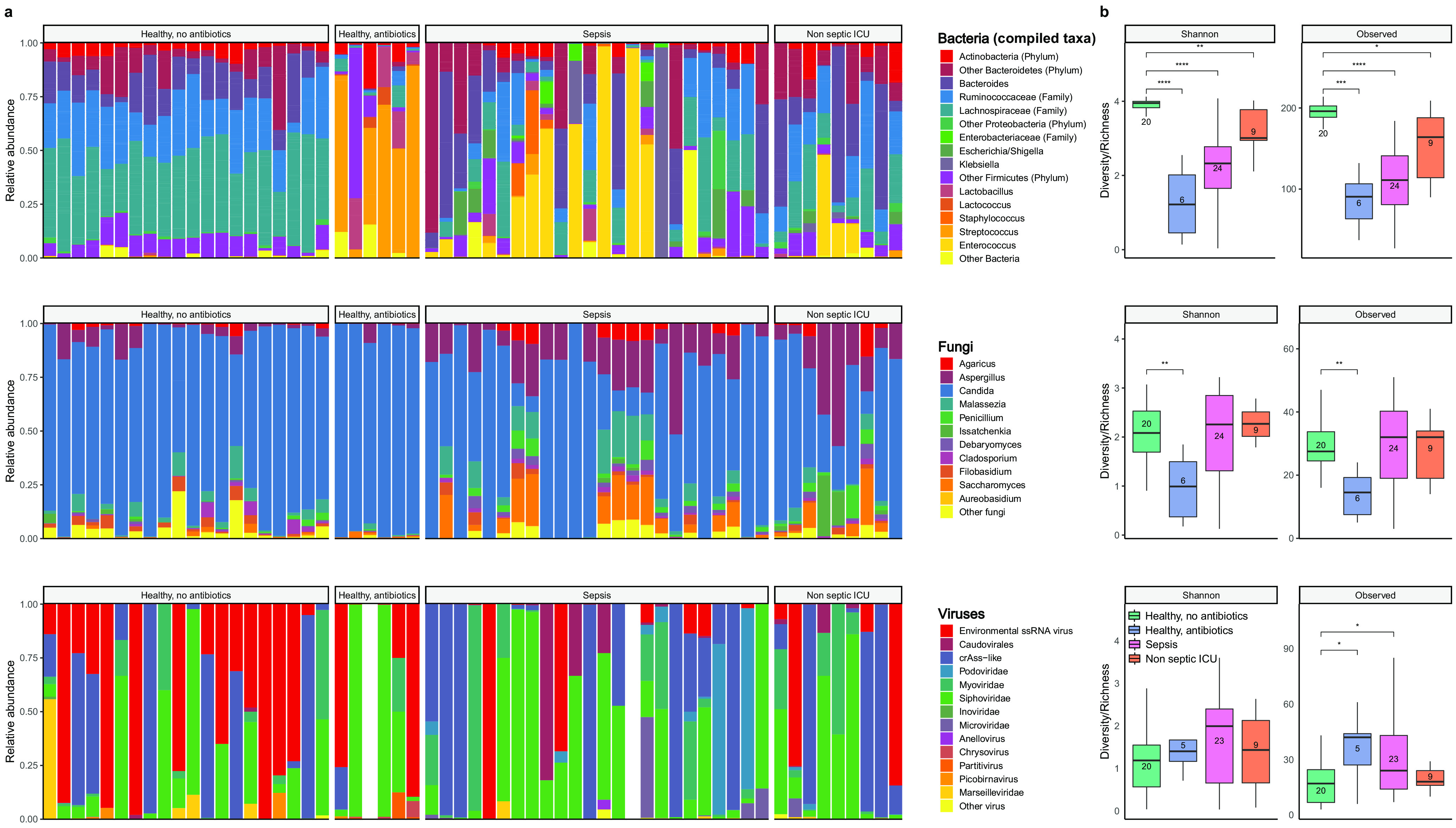
Overview of the composition and diversity of the bacterial, fungal, and viral microbiome. (a) Relative proportions of sequence reads at the genus level assigned to different bacterial and fungal taxa and at the order level for viral taxa. Viral metagenomics of two samples did not pass quality control due to high background levels, and these samples were therefore excluded from further analysis. For bacteria, the *Ruminococcaceae*, *Lachnospiraceae*, and *Enterobacteriaceae* families as well as genera that made up ≥5% of the total microbiota in at least one sample are included; other genera and families are pooled within the category “Other Bacteria.” (b) Alpha diversity metrics of bacteria (top), fungi (middle), and viruses (bottom), using the Shannon diversity index (Shannon) and the observed taxon richness index (Observed). In the box plots, the central rectangle spans the first quartile to the third quartile (the interquartile range [IQR]), the central line inside the rectangle shows the median, and whiskers above and below the box indicate variability outside the upper and lower quartiles. Given the nonparametric nature of the data, *P* values were calculated using the Wilcoxon rank sum test. *, *P* < 0.05; **, *P* < 0.01; ***, *P* < 0.001; ****, *P* < 0.0001.

First, we characterized the changes of each microbiome kingdom before and after antibiotic treatment. While the bacterial microbiome of healthy volunteers prior to antibiotic exposure was predominated by the anaerobic bacterial families *Lachnospiraceae* and *Ruminococcaceae*, the bacterial composition of both ICU patients and volunteers following antibiotics was characterized by an individualized loss of these anaerobic communities (Fig. 1a). In addition, bacterial alpha diversity and richness dropped significantly in ICU patients and healthy subjects exposed to antibiotics, with the latter being most significantly impacted in both metrics (Fig. 1b). In line with previous observations (30–32), fungal communities were dominated by *Candida* and *Saccharomyces*, while *Malassezia* and *Aspergillus* were also frequently observed. Overall, fungal diversity metrics were comparable between ICU patients and healthy controls not exposed to antibiotics, while significant drops in diversity were observed in healthy subjects after exposure to antibiotics. Viral communities were largely dominated by environmental single-stranded RNA (ssRNA) viruses and bacteriophages of the order *Caudovirales*. Strikingly, around 50% of the abundance of the virome consisted of cross-assembly (crAss) phages, which have recently been connected to *Bacteroides* spp. (33, 34). No differences in viral alpha diversity were observed, yet both septic ICU patients and antibiotic-perturbed volunteers displayed higher viral richness. We observed short-term temporal stability of all three kingdoms in healthy subjects not receiving antibiotics (35) (Fig. S2). In agreement with recent studies (36, 37), we observed that a total of 30% of healthy subjects were colonized by the anaerobic gut protozoon Blastocystis hominis or Dientamoeba fragilis, yet these protozoa were undetectable following antibiotic administration (Table S2).

10.1128/mSystems.01148-20.2 FIG S2 Temporal stability of bacterial, fungal, and viral microbiomes in healthy volunteers. (a) Samples collected from healthy volunteers (*n* = 7) not exposed to antibiotics on day 0 and day 9. Relative proportions of sequence reads assigned to different bacterial, fungal, and viral taxa are shown. For bacteria, the *Ruminococcaceae*, *Lachnospiraceae*, and *Enterobacteriaceae* families as well as genera that made up ≥5% of the total microbiota in at least one sample are included; other genera and families are pooled within the category “Other Bacteria.” (b) Alpha diversity metrics of bacteria, fungi, and viruses, using the Shannon diversity index (Shannon) and the observed taxon richness index (Observed). In the box plots, the central rectangle spans the first quartile to the third quartile (the interquartile range [IQR]), the central line inside the rectangle shows the median, and whiskers above and below the box indicate variability outside the upper and lower quartiles. Given the nonparametric nature of the data, *P* values were calculated using the Wilcoxon rank sum test. Download FIG S2, TIF file, 1.2 MB. Copyright © 2021 Haak et al. 2021 Haak et al. https://creativecommons.org/licenses/by/4.0/ This content is distributed under the terms of the Creative Commons Attribution 4.0 International license.

10.1128/mSystems.01148-20.9 TABLE S2 Overview of the intestinal presence of gut protozoa in all included patients. Download Table S2, DOCX file, 0.01 MB. Copyright © 2021 Haak et al. 2021 Haak et al. https://creativecommons.org/licenses/by/4.0/ This content is distributed under the terms of the Creative Commons Attribution 4.0 International license.

### Multi-omics factor analysis reveals covariation patterns across kingdoms.

**FIG 2:**
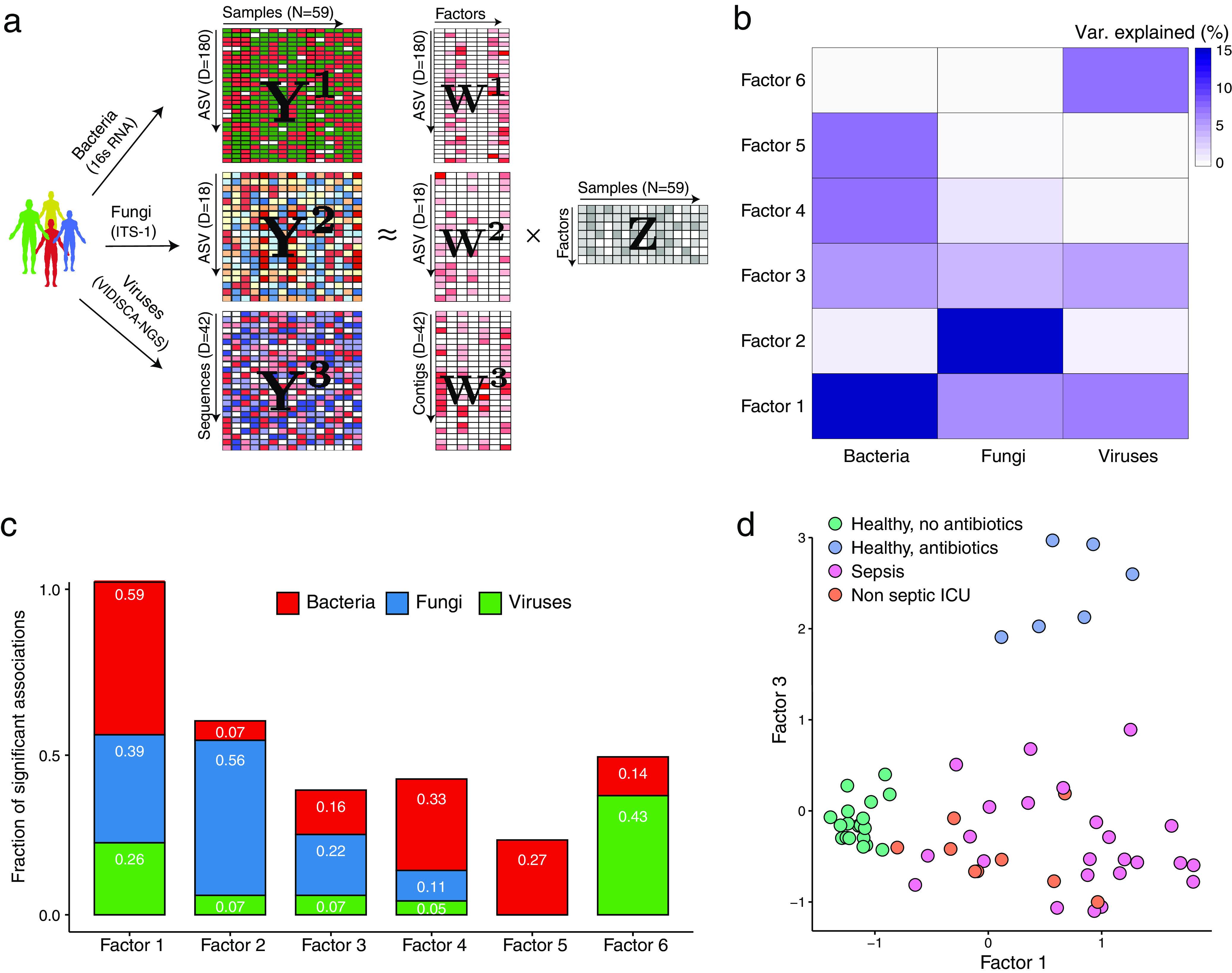
Multi-omics factor analysis (MOFA) delineates the sources of transkingdom heterogeneity. (a) Model overview. MOFA takes as the input the three microbiome quantification matrices. MOFA exploits the covariation patterns between the features within and between microbiome modalities to learn a low-dimensional representation of the data in terms of a small number of latent factors (Z matrix) and three different weight matrices (W) (one per kingdom). By maximizing the variance explained under sparsity assumptions, MOFA provides a principled way to discover the global sources of variability in the data. For each latent factor (i.e., each source of variation), the weights provide a measure of feature importance for every feature in each factor, hence enabling the interpretation of the variation captured by every factor. (b) Heat map displaying the percentage of variance explained (*R*^2^) by each factor (rows) across the three microbe modalities (columns). Factors 1 and 3 capture coordinated variation across all three microbiome modalities, whereas factor 2 is mostly dominated by heterogeneity in fungal composition. (c) Bar plots showing the fraction of significant associations between the features of each microbiome modality and each factor. *P* values were obtained using a *t* test based on Pearson’s product-moment correlation coefficient. Statistical significance is called at a 10% FDR. This plot is useful to interpret whether the variance-explained values displayed in panel b are driven by a strong change in a small number of features or by a moderate effect across a large range of features. (d) Scatterplot of factor 1 (*x* axis) versus factor 3 (*y* axis). Each dot represents a sample, colored by condition. Factor 1 captures the gradient in microbiome variation associated with antibiotic treatment and critical illness (from negative to positive factor values), whereas factor 3 captures the variation associated with antibiotic treatment in healthy patients (positive factor 3 values) versus critically ill patients (negative factor 3 values).

Next, to understand the patterns of covariation between these intestinal communities, we used multi-omics factor analysis (MOFA), a statistical framework for multi-omics data integration (38, 39). MOFA is a statistically rigorous generalization of principal-component analysis (PCA) to multi-omics data. Given multiple data modalities derived from the same sets of samples (Y matrices) (Fig. 2a), MOFA infers a common low-dimensional representation in terms of a small number of latent factors that capture the global sources of sample heterogeneity in the data (Z matrix) (Fig. 2a). Although the factors are inferred using information from all data modalities simultaneously, MOFA distinguishes variation that is shared across multiple modalities from the variation that is present within a single modality. In addition, MOFA facilitates the association of molecular features with each factor by the exploration of the feature weights (W matrices) (Fig. 2a). Notably, although this integrative method was initially conceived for single-cell multimodal assays (40), here, we show that it is also effective for the analysis of sparse readouts from microbiome data. For a more detailed mathematical treatment, see Materials and Methods.

As input into the model, we collapsed the inferred bacterial and fungal ASVs and viral reads to their respective family or genus level, followed by centralized log ratio normalization (41, 42). MOFA identified 6 factors with a minimum explained variance of 5% (see Materials and Methods) that were robust to downsampling analysis (Fig. 2a; Fig. S3). Altogether, the latent representation explained 39% of the sample heterogeneity for bacteria, 39% for fungi, and 19% for viral composition (Fig. 2b and c; Fig. S4). Notably, factor 1 and factor 3 (sorted by the total variance explained) captured covariation across all three kingdoms, allowing partitioning of microbiome compositions of critically ill patients from those of healthy subjects exposed to antibiotics and unexposed healthy subjects (Fig. 2d).

10.1128/mSystems.01148-20.3 FIG S3 Downsampling analysis of the MOFA factors. (a) Assessing the consistency of the factors when downsampling the number of samples in the data set. The *y* axis shows the Pearson correlation between the downsampled factors (sorted by variance explained) and the factors when using the full data set. The *x* axis shows the downsampling fraction, from 0.5 (half of the samples removed) to 1.0 (no samples removed). Circles denote the mean Pearson correlations, and the error bars show the corresponding standard deviations. (b) Correlation matrix of the MOFA model downsampled at 75% of samples (rows) versus the original MOFA model (columns). The diagonal structure indicates that all 10 factors are consistent in both models. Download FIG S3, TIF file, 0.4 MB. Copyright © 2021 Haak et al. 2021 Haak et al. https://creativecommons.org/licenses/by/4.0/ This content is distributed under the terms of the Creative Commons Attribution 4.0 International license.

10.1128/mSystems.01148-20.4 FIG S4 Cumulative variance explained (per microbiome modality) (*y* axis) versus factor number (*x* axis). The dashed line indicates the number of factors that were selected for downstream analysis (minimum of 5% variance explained across all data modalities). Download FIG S4, TIF file, 0.1 MB. Copyright © 2021 Haak et al. 2021 Haak et al. https://creativecommons.org/licenses/by/4.0/ This content is distributed under the terms of the Creative Commons Attribution 4.0 International license.

**FIG 3:**
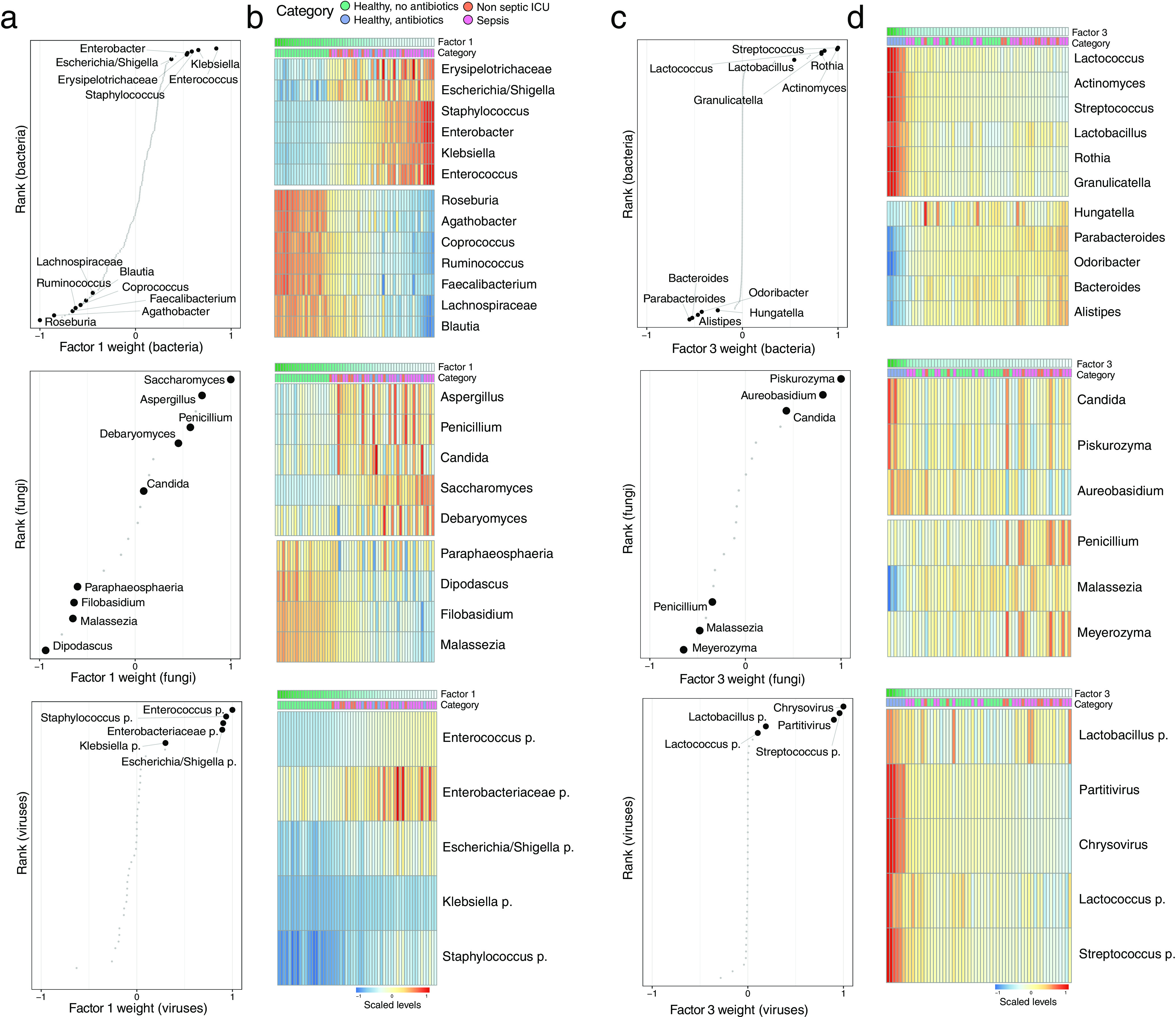
Characterization of the transkingdom covariation captured by factor 1 and factor 3. (a) Scatterplots displaying the distribution of bacterial (top), fungal (middle), and viral (bottom) weights for factor 1. A positive value indicates a positive association with factor 1 values, whereas a negative value indicates a negative association with factor 1 values (Fig. 2d). The larger the absolute value of the weight, the stronger the association. For ease of visualization, weights are scaled from −1 to 1. Representative taxa among the top weights are labeled. (b) Heat maps displaying the reconstructed data (see Materials and Methods) based on the MOFA model for the taxa highlighted in panel a. Samples are shown in the columns (sorted based on factor 1 values), and features are shown in the rows. (c) Scatterplots displaying the distribution of bacterial (top), fungal (middle), and viral (bottom) weights for factor 3. A positive value indicates a positive association with factor 3 values, whereas a negative value indicates a negative association with factor 3 values (Fig. 2d). The larger the absolute value of the weight, the stronger the association. For ease of visualization, weights are scaled from −1 to 1. Representative taxa among the top weights are labeled. (d) Heat maps displaying the (denoised) data reconstruction (see Materials and Methods) based on the MOFA model for the taxa highlighted in panel c. Samples are shown in the columns (sorted based on factor 3 values), and features are shown in the rows.

Factor 1, the major source of variation, was linked to a transkingdom signature driven by antibiotic perturbation in both health and critical illness (Fig. 3a and b). Specifically, bacterial taxa positively associated with this factor were facultative aerobic bacterial pathobionts that have been previously associated with critical illness (43–45), such as *Staphylococcus*, *Enterococcus*, *Klebsiella*, *Escherichia*/*Shigella*, and *Enterobacter*. Bacterial taxa that were negatively associated with this factor consisted predominantly of genera within the obligatory anaerobic families *Lachnospiraceae* and *Ruminococcaceae*, which have been identified as markers of a healthy microbiota and are linked to colonization resistance against bacterial pathobionts (10, 46). Fungal taxa positively associated with this factor were characterized by yeasts capable of causing invasive disease, such as *Candida*, *Aspergillus*, and *Debaryomyces* (24, 47, 48), with a relative absence of the gut constituents *Filobasidium*, *Malassezia*, and *Dipodascus* (31). The specific cooccurrences of fungal and bacterial taxa observed in factor 1 are supported by previous studies. For example, members of the *Lachnospiraceae* family, such as *Blautia* and *Roseburia*, display a direct inhibitory effect on the growth of several *Candida* spp. and Saccharomyces cerevisiae through the production of short-chain fatty acids (SCFAs) and other metabolites (49–51). In addition, *in vitro* studies have shown that metabolites produced by *Candida* spp. enhance the growth of Escherichia coli and Staphylococcus aureus (52, 53), providing further indications that the *in vivo* intestinal transkingdom signatures identified by MOFA are biologically meaningful.

Factor 3 captured signatures that were absent in critically ill patients and present in healthy subjects following exposure to broad-spectrum antibiotics, with a predominance of the closely related *Streptococcaceae* family (*Streptococcus* and *Lactococcus*), *Lactobacillales* order (*Lactobacillus* and *Granulicatella*), and *Actinomycetales* order (*Actinomyces* and *Rothia*). While 16S rRNA sequencing provides limited resolution to identify the ASVs on the species and strain levels, several species of these bacterial orders and families have been shown to possess mutualistic properties with *Candida* in oral and vaginal environments, potentially through the modification of biofilm formation (23, 54–56). These observations indicate that similar fungal-bacterial associations are potentially maintained within the gastrointestinal tract, warranting further elucidation on a larger scale and at a higher taxonomic resolution.

Notably, the majority of viral sequences that were associated with factors 1 and 3 consisted of bacteriophages that significantly correlated with the presence of the corresponding bacterial targets in the same factor (Fig. S5). The expansion of aerobic bacterial species during critical illness and following antibiotics can therefore potentially facilitate the enrichment of their corresponding bacteriophages (12, 57). Other notable associations with the viral microbiome were the increases of the mycoviruses *Chrysovirus* and *Partitivirus*, which are capable of infecting fungi (58), in healthy subjects following antibiotic exposure. These findings indicate that coordinated transkingdom changes are occurring beyond intestinal bacteria, further underscoring the complexity of relationships within the intestinal environment.

10.1128/mSystems.01148-20.5 FIG S5 Positive association between bacterial levels and associated viral phages. (a) Scatterplots of the viral weights (*x* axis) versus bacterial weights (*y* axis) for MOFA factor 1 (left) and factor 3 (right). Each dot corresponds to a bacterium and its associated phage. (b) Scatterplots of the concentrations of bacteria (*x* axis) and the corresponding viral phages (*y* axis). Each dot corresponds to one sample and is colored by category (healthy no antibiotic, healthy antibiotic, sepsis, and ICU). In all figures, the line depicts the linear regression fit, and the shading depicts the corresponding 95% confidence interval. Pearson correlation coefficients and associated *P* values are displayed in the top left corner. Download FIG S5, TIF file, 1.0 MB. Copyright © 2021 Haak et al. 2021 Haak et al. https://creativecommons.org/licenses/by/4.0/ This content is distributed under the terms of the Creative Commons Attribution 4.0 International license.

After the global characterization of the transkingdom microbiome associations upon antibiotic exposure, we asked whether we could find associations between individual MOFA factors and specific antibiotics. Whereas factor 1 is associated with exposure to antibiotics in general, factor 4 was specifically associated with exposure to fluoroquinolones and negatively correlated with exposure to cephalosporins (Fig. S6a and b). Specifically, we observed that patients receiving fluoroquinolones had higher abundances of the Gram-positive genera *Lactococcus* and *Pediococcus* and lower abundances of, among others, the Gram-negative genera *Escherichia*/*Shigella* and *Desulfovibrio*. Interestingly, we observed a negative correlation between *Aspergillus* and fluoroquinolone exposure, which could be linked to the previously described synergy between ciprofloxacin and antifungal agents directed toward *Aspergillus* (59).

10.1128/mSystems.01148-20.6 FIG S6 Characterization of factor 4 as a microbiome response to cephalosporins and quinolones. (a) Analysis of the association between factors and antibiotic treatment. (Top) Pearson correlation coefficients between factor values and the antibiotic treatment indicator variables. (Bottom) Associated log_10_ FDR-adjusted *P* values. (b) Beeswarm and violin plots displaying factor 4 values, colored by cephalosporin (left) and quinolone (right) treatment. (c) Distribution of bacterial (left) and fungal (right) weights for factor 4. Labeled are representative bacteria and fungi among the largest weights. (d) Heat map displaying the reconstructed and scaled ASV levels (see Materials and Methods) for the bacteria labeled in panel c. (e) Scatterplot displaying the association between factor values and normalized ASV levels in *Aspergillus*. The line represents the linear regression fit, and the shading represents the corresponding 95% confidence interval. Pearson correlation coefficients and associated *P* values are displayed in the top left corner. Download FIG S6, TIF file, 2.1 MB. Copyright © 2021 Haak et al. 2021 Haak et al. https://creativecommons.org/licenses/by/4.0/ This content is distributed under the terms of the Creative Commons Attribution 4.0 International license.

The 3 remaining factors identified sample heterogeneity related to low-abundance fungal variations (factor 2) (Fig. S7) as well as bacterial (factor 5) and viral (factor 6) signatures pertaining to individual ICU patients.

10.1128/mSystems.01148-20.7 FIG S7 Depiction of factor 2, characterized by variation of low-abundance fungi in critical illness. (a) Beeswarm and violin plots displaying factor 2 values, indicating that the factor is driven by features that are predominantly present or absent during critical illness, with limited involvement within healthy volunteers. (b) Distribution of fungal weights for factor 2. Labeled are representative fungi among the largest weights. (c) Heat map displaying the reconstructed and scaled ASV levels (see Materials and Methods) for the fungi labeled in panel b. (d) Scatterplot displaying the association between factor values and normalized ASV levels. The line represents the linear regression fit, and the shading represents the corresponding 95% confidence interval. Pearson correlation coefficients and *P* values are displayed. Download FIG S7, TIF file, 1.9 MB. Copyright © 2021 Haak et al. 2021 Haak et al. https://creativecommons.org/licenses/by/4.0/ This content is distributed under the terms of the Creative Commons Attribution 4.0 International license.

### Fecal levels of short-chain fatty acids are negatively correlated with fungal loads in health and critical illness.

**FIG 4:**
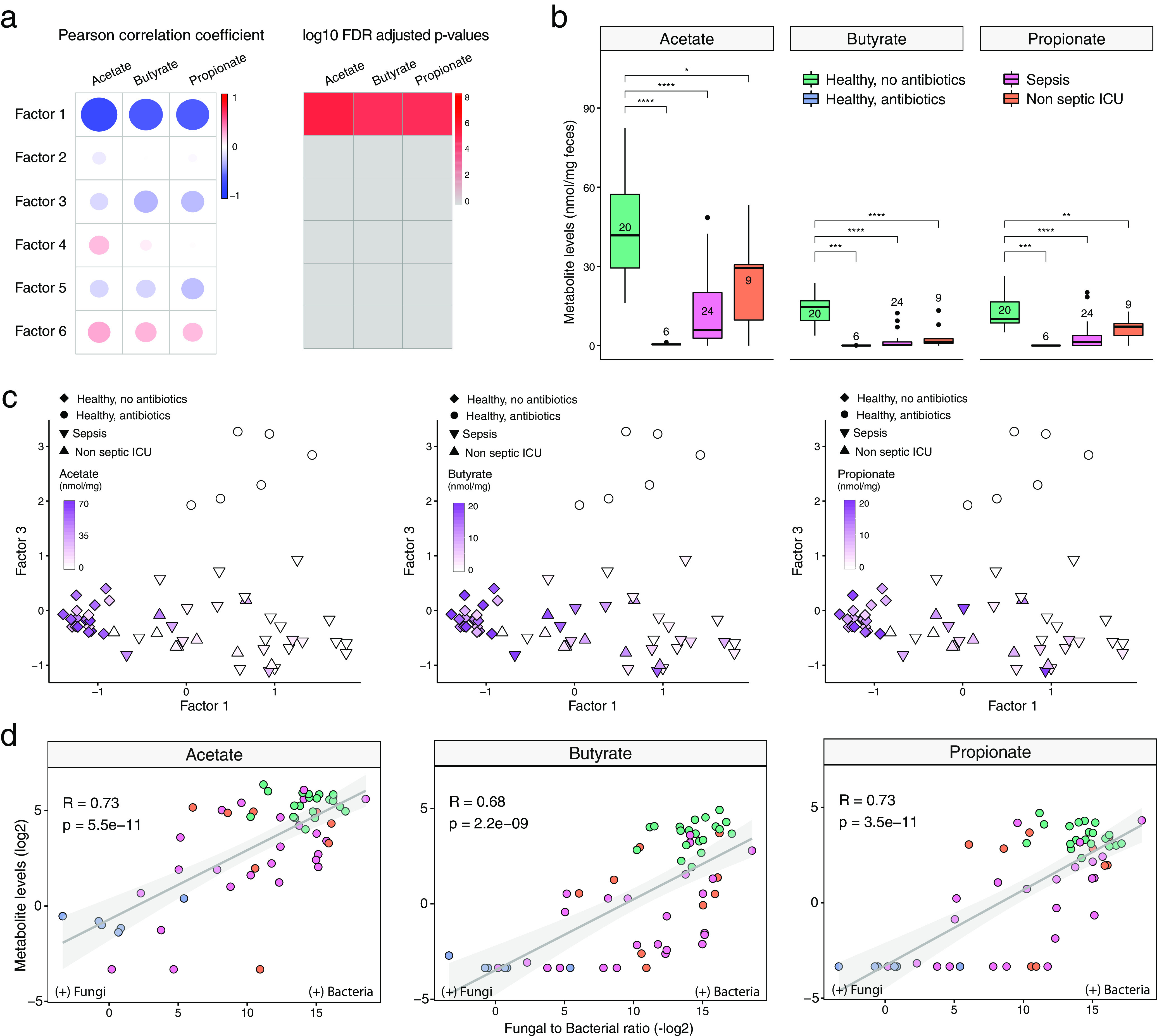
Correlation of total bacterial and fungal loads with fecal levels of short-chain fatty acids in health and critical illness. (a) Association analysis between factor values and SCFA levels. (Left) Pearson correlation coefficients between factor values and the levels of three types of SCFAs: butyrate, acetate, and propionate. (Right) Corresponding FDR-adjusted and log-transformed *P* values. (b) Box plots showing the SCFA concentrations (*y* axis) per sample group (*x* axis). In the box plots, the central rectangle spans the first quartile to the third quartile (the interquartile range [IQR]), the central line inside the rectangle shows the median, and whiskers above and below the box indicate variability outside the upper and lower quartiles. Given the nonparametric nature of the data, *P* values were calculated using the Wilcoxon rank sum test. *, *P* < 0.05; **, *P* < 0.01; ***, *P* < 0.001; ****, *P* < 0.0001. (c) Scatterplot of factor 1 (*x* axis) versus factor 3 (*y* axis) values. Each dot represents a sample, shaped by the sample group and colored by SCFA concentrations (in milligrams per milligram of feces). (d) Scatterplot of fungal-to-bacterial absolute level ratios (after log_10_ transformation) (*x* axis) versus SCFA concentrations (after log_2_ transformation) (*y* axis). The line represents the linear regression fit, and the shading represents the corresponding 95% confidence interval. Corresponding Pearson correlation coefficients and *P* values are also displayed in the top left corner.

An important indicator of the influence of the bacterial microbiota on the fungal population in the gut is the dramatic increase in the fungal burden after antibiotic treatment (23). This phenomenon can partly be explained by antibiotic-induced alterations in nutrient availability, yet a loss of the direct inhibitory effects of anaerobic bacteria and their associated metabolites toward fungal expansion has also been documented (49–51). In light of these observations, we quantified absolute levels of bacteria and fungi using targeted PCRs and linked their abundance to the absolute quantities of the SCFAs butyrate, propionate, and acetate, which are well-known metabolites of predominantly anaerobic bacteria. First, we observed a strong depletion of SCFAs both in critical illness and following antibiotic perturbation (Fig. 4a to c). Notably, both conditions were associated with increased fungal-to-bacterial ratios, with the relative proportion of fungi to bacteria increasing by a factor of 10^3^ to 10^4^ times. In addition, we observed that absolute fecal SCFA concentrations were inversely correlated with absolute fungal copies, with propionate levels displaying the strongest correlation (*r* = 0.75; *P* < 0.0001) (Fig. 4d). These findings are in line with those of a recent study reporting that a reduction of anaerobic bacteria during the course of allogeneic hematopoietic stem cell transplantation directly facilitates the intestinal overgrowth of specific *Candida* species, ultimately culminating in invasive fungal disease (24). Therefore, our findings and those of others suggest that fungal expansion not only occurs in the context of a decreased absolute bacterial abundance but also is dependent on altered functions of the remaining bacterial communities in the intestinal environment.

### Perspectives and limitations.

This study has several limitations. First, since 16S rRNA sequencing provides limited taxonomic resolution of bacterial communities at the species level, data were collapsed to the genus level. While our findings remained robust in a downsampling analysis, implementing MOFA with larger data sets with higher resolution, such as those obtained by shotgun metagenomic sequencing, will be an important next step to further uncover the more intricate transkingdom cooccurrence associations, including interactions. Second, this study mainly describes associations between bacteria, fungi, and viruses, but it does not directly prove causality that these shifts are mechanically linked. However, our findings are in line with those of several preclinical *in vitro* studies, providing assurance that these transkingdom effects seem to also occur in humans. Third, although our study is among the first to validate several preclinical findings in humans and emphasizes the importance of transkingdom disruptions of the intestinal microbiome during critical illness, it was not powered to detect if the observed disruptions were associated with altered outcomes such as increased mortality or length of hospital stay. Finally, while the characterization of demographic information and antibiotic exposure in our cohorts was excellent, our analyses did not account for the different times between antibiotic exposure and sample collection nor for other well-known confounders such as dietary habits, (par)enteral feeding, and exposure to (vasoactive) drugs.

In conclusion, our findings shed light on the covariation patterns between kingdoms following broad-spectrum antibiotic modulation, both in health and in the context of critical illness. These findings underscore the potential value of multi-omics data integration tools in interrogating how different components of the microbiota contribute to health and disease.

## MATERIALS AND METHODS

### Study design and participants.

Patients were recruited as part of a large prospective observational study in critically ill patients admitted to the ICU (Molecular Diagnosis and Risk Stratification of Sepsis [MARS] study [ClinicalTrials.gov identifier NCT01905033]) (6, 60). A total of 33 randomly selected adult patients who were admitted to the ICU of the Academic Medical Center (Amsterdam, The Netherlands) between October 2012 and November 2013 were included. Patients who were transferred from other ICUs or had an expected length of ICU stay of <24 h were excluded. All patients met at least two of the following criteria: body temperature of ≤36°C or ≥38°C, tachycardia of >90 beats/min, tachypnea of >20 breaths/min or partial pressure of carbon dioxide (pCO_2_) of <4.3 kPa, and leukocyte count of <4 × 10E9 leukocytes/liter or >12 × 10E9 leukocytes/liter. Sepsis was defined when the inclusion criteria were associated with suspected infection within 24 h after ICU admission, with subsequent systemic therapeutic administration of antibiotics to the patient (6). The control group consisted of 13 healthy, nonsmoking, Caucasian male subjects (aged 18 to 25 years; mean age, 22 years) who had not taken antibiotics during the previous year (ClinicalTrials.gov identifier NCT02127749) (29, 61). Six healthy subjects received oral broad-spectrum antibiotics (ciprofloxacin at 500 mg q12h, vancomycin at 500 mg q8h, and metronidazole at 500 mg q8h) for 7 days. Subjects were asked to collect fecal samples before antibiotic treatment and 1 day after the 7-day course of antibiotics. Fresh stool samples from ICU patients were stored at 4°C and transferred to −80°C within 24 h of collection. Fecal samples from healthy subjects were collected in plastic containers, stored at −20°C at home, and transported to the study center for storage at −80°C within 24 h.

### Bacterial and fungal microbiota sequencing.

Fecal DNA was extracted and purified using a combination of repeated bead beating (method 5) (62) and the Maxwell 16 tissue Low Elution Volume total RNA purification kit (Promega, Madison, WI, USA), with STAR (stool transport and recovery) buffer (Roche, Basel, Switzerland). Negative extraction controls (DNA-free water) were processed in a similar manner.

Twenty nanograms of DNA was used for the amplification of the bacterial 16S rRNA gene with the V3-V4 341F forward primer and the 805R reverse primer for 25 cycles. The PCR was performed in a total volume of 30 μl containing 1× High Fidelity buffer (Thermo Fisher Scientific, Waltham, MA, USA); 1 μl deoxynucleoside triphosphate (dNTP) mix (10 mM; Promega, Leiden, The Netherlands); 1 U of Phusion green high-fidelity DNA polymerase (Thermo Fisher Scientific, Waltham, MA, USA); 500 nM the forward 8-nucleotide (nt) sample-specific barcode primer containing the Illumina adapter, pad, and link (341F [5′-CCTACGGGNGGCWGCAG-3′]); 500 nM the reverse 8-nt sample-specific barcode primer containing the Illumina adapter, pad, and link (805R [5′-GACTACHVGGGTATCTAATCC-3′]); 20 ng/μl of template DNA; and nuclease-free water. The amplification program was as follows: an initial denaturation step at 98°C for 30 s; 25 cycles of denaturation at 98°C for 10 s, annealing at 55°C for 20 s, and elongation at 72°C for 90 s; and an extension step at 72°C for 10 min (63). The size of the PCR products (∼540 bp) was confirmed by gel electrophoresis using 4 μl of the amplification reaction mixture on a 1% (wt/vol) agarose gel containing ethidium bromide (AppliChem GmbH, Darmstadt, Germany).

Fungal composition was determined by ITS1 amplicon sequence analysis. PCR-generated amplicon libraries were obtained from 100 ng fecal DNA using the ITS1 primer set containing an overhang for the Illumina Nextera platform (forward primer 5′-TCGTCGGCAGCGTCAGATGTGTATAAGAGACAGCTTGGTCATTTAGAGGAAGTAA and reverse primer 5′-GTCTCGTGGGCTCGGAGATGTGTATAAGAGACAGGCTGCGTTCTTCATCGATGC) and Phusion high-fidelity DNA polymerase (Thermo Fisher Scientific, Waltham, MA, USA). A duplicate reaction in 20 μl was performed with following thermocycling conditions: an initial denaturation step at 98°C for 1 min followed by 35 cycles of denaturation (20 s), annealing (20 s at 58°C), and extension (60 s at 72°C) and a final extension step at 72°C for 5 min. The duplicates were pooled to a final volume of 40 μl. The PCR products were purified with AMPure XP beads (Beckman Coulter, Brea, CA, USA) and taken into 15 μl DNA-free water. A second amplification step was used to introduce multiplex indices and the Illumina sequencing adapters using the Kapa polymerase system. The reaction was performed in 40 μl using the following thermocycling conditions: initial denaturation at 95°C for 3 min followed by 24 cycles of denaturation (20 s at 98°C), annealing (20 s at 60°C), and extension (60 s at 72°C) and a final extension step at 72°C for 5 min.

Bacterial and fungal PCR products were purified using AMPure XP beads (Beckman Coulter, Brea, CA, USA). The amplicon DNA concentration was measured using the Qubit fluorometric quantitation method (Thermo Fisher Scientific, Waltham, MA, USA), and DNA quality was determined using the Agilent Bioanalyzer DNA-1000 chip, after which the purified products were equimolarly pooled. The libraries were sequenced in a paired-end run with 251 cycles on an Illumina MiSeq platform (GATCBiotech, Constance, Germany) using V3 chemistry. Forward and reverse reads were truncated to 240 and 210 bases, respectively, and merged using USEARCH (64). Merged reads that did not pass the Illumina chastity filter, had an expected error rate higher than 2, or were shorter than 380 bases were filtered. Amplicon sequencing variants (ASVs) were inferred for each sample individually with a minimum abundance of 4 reads (25). Unfiltered reads were then mapped against the collective ASV set to determine the abundances. Bacterial taxonomy was assigned using the RDP classifier (65) and SILVA 16S ribosomal database V132 (66). Fungal taxonomy was assigned using the UNITE database (67). Of note, for the purpose of this study, the bacterial microbiomes of ICU patients (6) and volunteers (29) were resequenced together to reduce batch effects.

### Viral microbiota sequencing and analysis.

The collected fecal suspension was centrifuged to pellet cells and debris, and nucleic acids in the supernatant were extracted using the Boom method (68), followed by reverse transcription with nonribosomal random hexamers (69) and second-strand synthesis. DNA was digested with MseI (T^TAA; New England BioLabs, Ipswich, MA, USA) and ligated to adapters containing a sample identifier sequence. Next, size selection with AMPure XP beads (Beckman Coulter, Brea, CA, USA) was performed to remove small DNA fragments prior to a 28-cycle PCR using adapter-annealing primers. Small and large size selection was performed with AMPure XP beads to select DNA strands with a length ranging between 150 and 550 nucleotides. Libraries were analyzed using the Bioanalyzer (high-sensitivity [HS] kit; Agilent Genomics, Santa Clara, CA, USA) and Qubit (dsDNA [double-stranded DNA] HS assay kit; Thermo Fisher Scientific, Waltham, MA, USA) instruments to quantify the DNA length and concentration, respectively. Sample libraries were pooled at equimolar concentrations. In total, 50 pmol DNA of the pool was clonally amplified on beads using the Ion Chef system (Thermo Fisher Scientific, Waltham, MA, USA), and sequencing was performed on the Ion PGM system (Thermo Fisher Scientific, Waltham, MA, USA) with the Ion 316 chip (400-bp read length and 2 million sequences expected per run).

VIDISCA-NGS reads were aligned using BWA-MEM (70) to a reference database consisting of the human reference genome (hg38), the SILVA SSU V132 database (66), and all RefSeq viral genomes (downloaded in September 2019). Mapping outputs were further processed using the PathoID module of PathoScope 2.0 (71, 72) to reassign reads with multiple alignments to their most likely target. Viral candidates were aligned back to the reference database with BLASTn, and those aligning at ≥95% for 100 bp were retained as hits. To ensure that all known eukaryotic viruses were detected with this approach, all reads that remained unmapped in the BWA-MEM step were analyzed with a separate virus discovery bioinformatic pipeline described in detail previously (28). Briefly, rRNA reads were identified with SortMeRNA v2.1, non-rRNA reads were made nonredundant using CD-HIT v4.7, and these were queried against a eukaryotic virus protein database using the UBLAST algorithm provided as part of the USEARCH v10 software package (64). Reads with a significant alignment to a viral protein were subsequently aligned to the nonredundant nucleotide database using BLASTn. Those with a best hit to a viral sequence were regarded as confidently viral, and those not aligning to any sequences were regarded as putatively viral, while those with a nonviral best hit were regarded as false positives.

### Targeted measurement of intestinal protozoa.

Automated nucleic acid extraction was performed on the MagNA Pure 96 instrument (Roche Applied Science, The Netherlands) according to the manufacturer’s protocol. DNA was eluted in a 100-μl elution buffer (Roche Applied Science). Phocine herpesvirus (PhoHV) DNA was added to all samples as an internal control for extraction and amplification efficiency. The presence of Giardia lamblia, Cryptosporidium parvum, Entamoeba histolytica, *Blastocystis hominis*, and *Dientamoeba fragilis* was assessed by real-time PCR targeting the small-subunit rRNA gene (37). Positive controls consisting of a plasmid containing the target sequence were included in every run, as were negative extraction controls and negative PCR controls. Subjects were excluded from further analyses if internal controls tested negative in one or more samples.

### Targeted measurement of short-chain fatty acids.

Sample preparation of fecal extracts and nuclear magnetic resonance (NMR) spectroscopy for the quantification of SCFAs were performed as described previously by H. K. Kim et al. (73), with some modifications. Briefly, aqueous extracts of feces were prepared by mixing 50 to 100 mg of feces and 0.3 ml of deionized water, followed by mechanical homogenization in a Bullet Blender 24 (Next Avance Inc., Troy, NY, USA). The fecal slurry was centrifuged twice at 18,213 × *g* for 10 min at 4°C, and 0.225 ml of the supernatant was mixed with 0.025 ml of 1.5 M potassium phosphate buffer (pH 7.4) containing 2 mM sodium azide and 4 mM sodium trimethylsilyl-propionate-d_4_ (TSP-d_4_) in D_2_O. For each sample, the one-dimensional (1D) ^1^H-NMR spectrum was acquired in a 14.1 T Avance II NMR instrument (Bruker Biospin Ltd., Karlsruhe, Germany). Quantification of SCFAs from the NMR spectra was performed in ChenomX (Chenomx NMR suite 8.4) using the known concentration of TSP-d_4_.

### Quantitative PCR for bacterial and fungal loads.

For the measurement of the total bacterial content in fecal samples, we used a method reported previously by Nadkarni and colleagues (74), with modifications. Briefly, we used a primer concentration of 500 nM in a final volume of 10 μl with the SensiFast SYBR No-ROX kit (Bioline, London, UK). The amplification conditions were as follows: an initial denaturation step at 95°C for 5 s followed by denaturation (10 s at 95°C), annealing (10 s at 66°C), and extension (20 s at 72°C) for 44 repetitive cycles in a Bio-Rad (Hercules, CA, USA) CFX96 thermocycler. The primer set of FungiQuant (75) was used for fungal load determination, with modifications. The final PCR primer concentration was 500 nM in a volume of 10 μl with the SensiFast SYBR No-ROX kit (Bioline, London, UK). The following amplification program was used: an initial denaturation step at 95°C for 5 s followed by denaturation (10 s at 95°C), annealing (10 s at 60°C), and extension (20 s at 72°C) in 44 repetitive cycles in a Bio-Rad (Hercules, CA, USA) CFX96 thermocycler. Following amplification, fungal and bacterial ratios were calculated using LinRegPCR (76).

### Multi-omics factor analysis: model description.

The input into multi-omics factor analysis (MOFA) is a set of *M* data matrices, Y^1^,…, Y*^M^*, of dimensions *N* × *D_m_*, where *N* is the number of samples and *D_m_* is the number of features in data matrix *m*. MOFA decomposes these matrices as

$$Y^{m}=ZW^{\mathrm{mT}}+\varepsilon^{m}\;\;\;\;\;m=1,\ldots ,\;M$$

Here, Z denotes the factor matrix and W*^m^* denotes the weight matrices for each data modality *m*. ε*^m^* denotes the modality-specific Gaussian noise term ${\text{ε}}_{nd}^{m}˜N(0,1/{\text{τ}}_{d}^{m})$.

The model is formulated in a probabilistic Bayesian framework, where prior distributions are placed on all unobserved variables of the model. The most important prior is the sparsity-inducing prior in the weights. MOFA uses a two-level regularization. The first level encourages modality- and factor-wise sparsity, thereby allowing the direct identification of which factor is active in which data modality. The second level encourages feature-wise sparsity, resulting in a small number of features with nonzero weights for each factor. Mathematically, MOFA encodes this by combining an automatic relevance determination prior for the first type of sparsity and a spike-and-slab prior for the second. Model inference is performed using variational Bayesian inference with mean-field assumption. We refer the reader to a previous publication from our group for further mathematical details (38).

### Multi-omics factor analysis: downstream analysis.

After model fitting, the number of factors was estimated by requiring a minimum of 5% variance explained across all microbiome modalities. The downstream characterization of the model output included several analyses:

- variance decomposition. The fraction of variance explained (*R*^2^) by each factor in each view is quantified using a coefficient of determination (38–40),
  $$R_{m,k}^{2}=1-{\left(\underset{n,d}{\Sigma }y_{nd}^{m}\;-\;z_{nk}w_{kd}^{m}\;-{\text{μ}}_{d}^{m}\right)}^{2}/{\left(\underset{n,d}{\Sigma }y_{nd}^{m}\;-{\text{μ}}_{d}^{m}\right)}^{2}$$
- visualization of weights. The model learns a weight for every feature in each factor, which can be interpreted as a measure of feature importance. Larger weights (in absolute values) indicate a higher correlation with the corresponding factor values. The sign of the weight indicates the directionality of the variation: features with positive weights are positively associated with the corresponding values, whereas features with negative weights are negatively associated with the corresponding values.
- visualization of factors. Each MOFA factor captures a different dimension of heterogeneity in the microbiome composition. Mathematically, each factor ordinates cells along a one-dimensional axis centered at zero. Samples with different signs manifest opposite phenotypes along the inferred axis of variation, with a higher absolute value indicating a stronger effect. Note that the interpretation of factors is analogous to the interpretation of the principal components in PCA.
- denoising by data reconstruction. MOFA generates a compressed low-dimensional representation of the data. By taking the product of the factors and the weights, the model can reconstruct a normally distributed denoised representation of the input data. This is particularly useful for the visualization of sparse readouts.

### Data processing for MOFA.

The input into MOFA is a set of data modalities with matching samples. In this case, bacterial 16S rRNA ASVs, fungal ITS1 rRNA ASVs, and viral sequences were defined as separate data modalities. As a filtering criterion, bacterial and fungal features were required to have a minimum of 10 ASVs observed in at least 25% of the data set. In addition, to mitigate the sparsity of the data and to simplify the interpretation, we collapsed the inferred bacterial and fungal ASVs and viral sequences to their respective family or genus level. The numbers of sequences were subsequently scaled using a centralized log ratio (41), which has been shown to be effective in normalizing compositional data (42).

### Significance of MOFA factors and downsampling analysis.

The significance of the MOFA factors can be assessed by the variance-explained estimates (per data modality) that result from the variance decomposition analysis. In addition, we performed a sensitivity analysis to quantify the robustness of the factors after downsampling the number of samples in the data set. For each downsampled version of the data, we have retrained a MOFA model, and for each model, we then correlated the resulting factors with the factors that are found with the full data set (see Fig. S3 in the supplemental material).

### Statistics.

All analyses were performed in the R statistical framework (version 3.6.1; R Foundation for Statistical Computing, Vienna, Austria). To assess alpha diversity and richness, we calculated the Shannon diversity index and the observed taxon richness index with the phyloseq package (17). Data were not normally distributed and are therefore presented as medians and interquartile ranges (IQRs), while data were analyzed using a Wilcoxon rank sum test. Associations between factor values and covariates were analyzed using linear regression by Pearson correlation coefficients. Statistical significance is called at a 10% false discovery rate (FDR).

### Ethics approval and consent to participate.

Ethical approval for both the patient and healthy subject studies was received from the Medical Ethics Committee of the Academic Medical Center in Amsterdam, and all research was conducted in accordance with the Declaration of Helsinki. Written informed consent was obtained from all healthy subjects and patients or their legal representatives.

### Availability of data.

Raw sequencing data (bacterial and fungal ASVs and VIDISCA-NGS sequencing reads) were submitted to the European Nucleotide Archive (ENA) (accession number PRJEB37289).

All code used for analysis is available at https://github.com/bwhaak/MOFA_microbiome. Links to the processed data are included in the GitHub repository.
