# Supplementary figures and images for: Integrative Transkingdom Analysis of the Gut Microbiome in Antibiotic Perturbation and Critical Illness
Source: mSystems. 2021 Mar 16;6(2):e01148-20. doi: 10.1128/mSystems.01148-20 (PMC8546997; doi:10.1128/mSystems.01148-20)

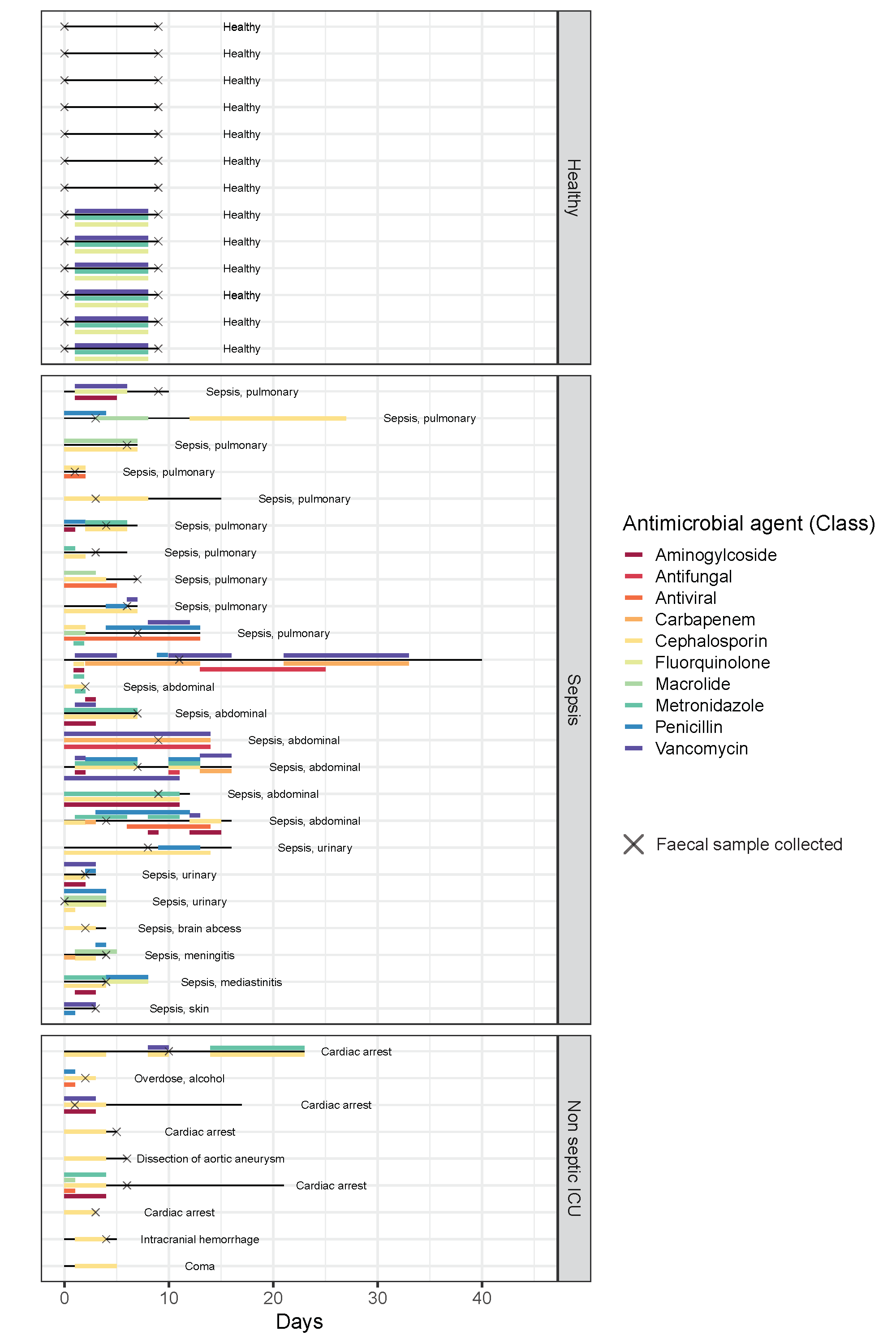

Supplement: FIG S1 [file msystems.01148-20-sf001.tif]

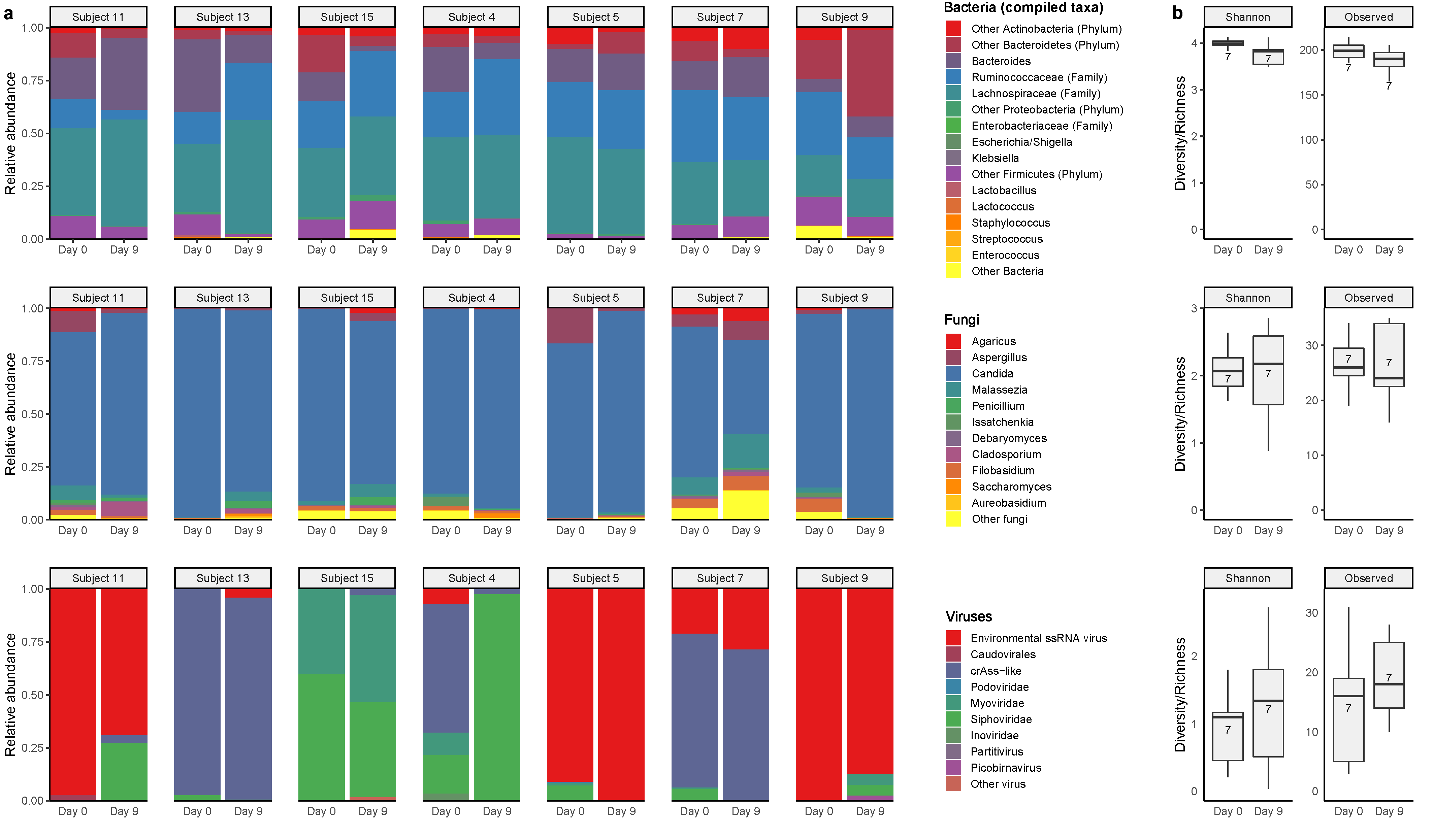

Supplement: FIG S2 [file msystems.01148-20-sf002.tif]

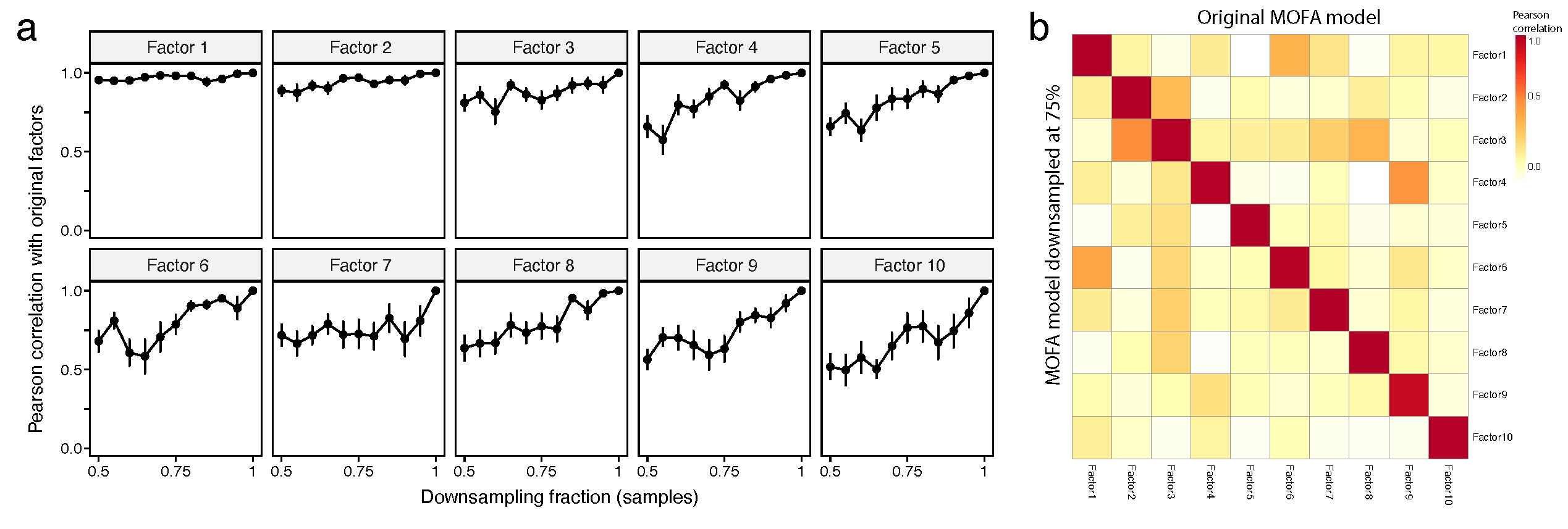

Supplement: FIG S3 [file msystems.01148-20-sf003.tif]

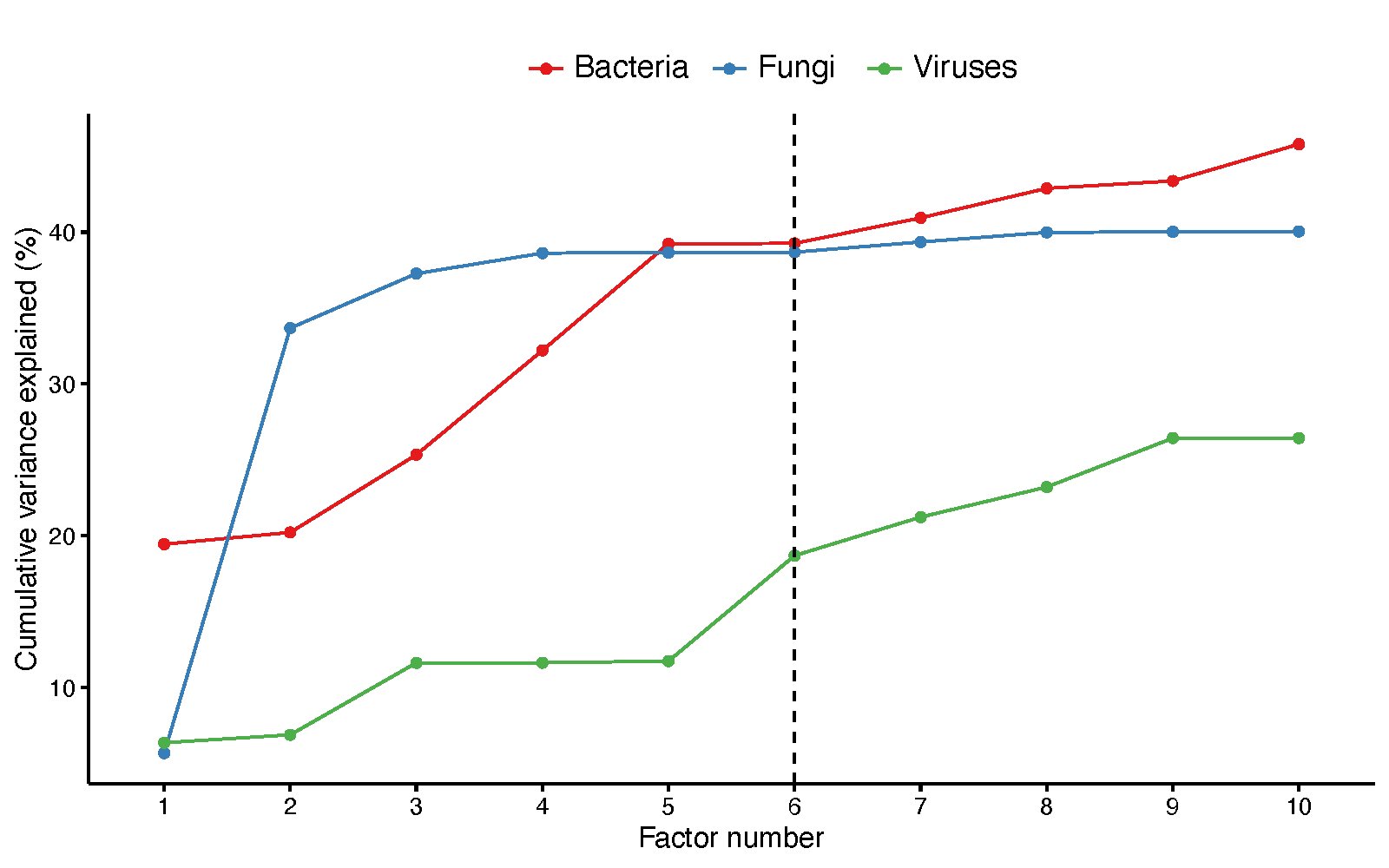

Supplement: FIG S4 [file msystems.01148-20-sf004.tif]

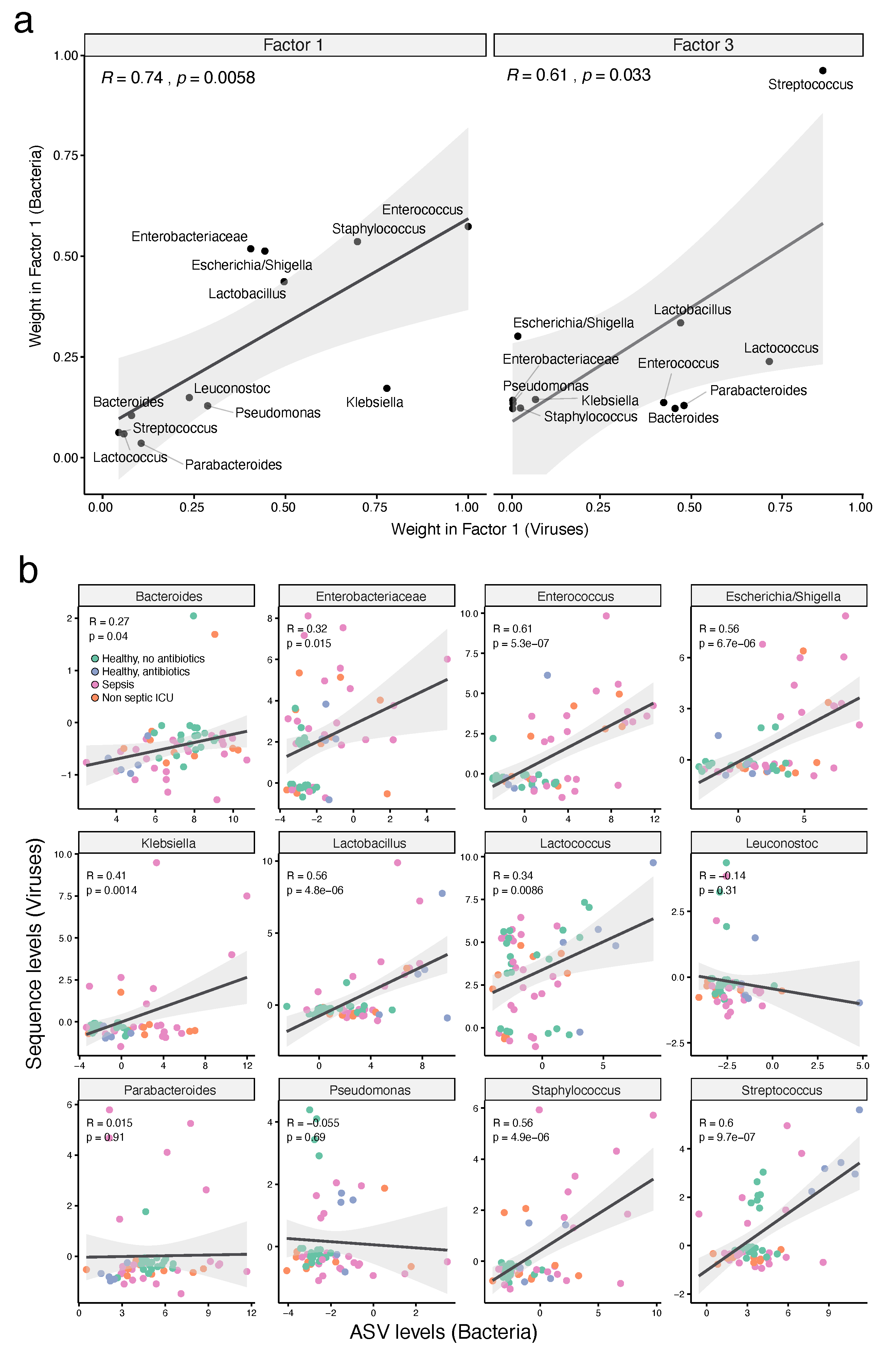

Supplement: FIG S5 [file msystems.01148-20-sf005.tif]

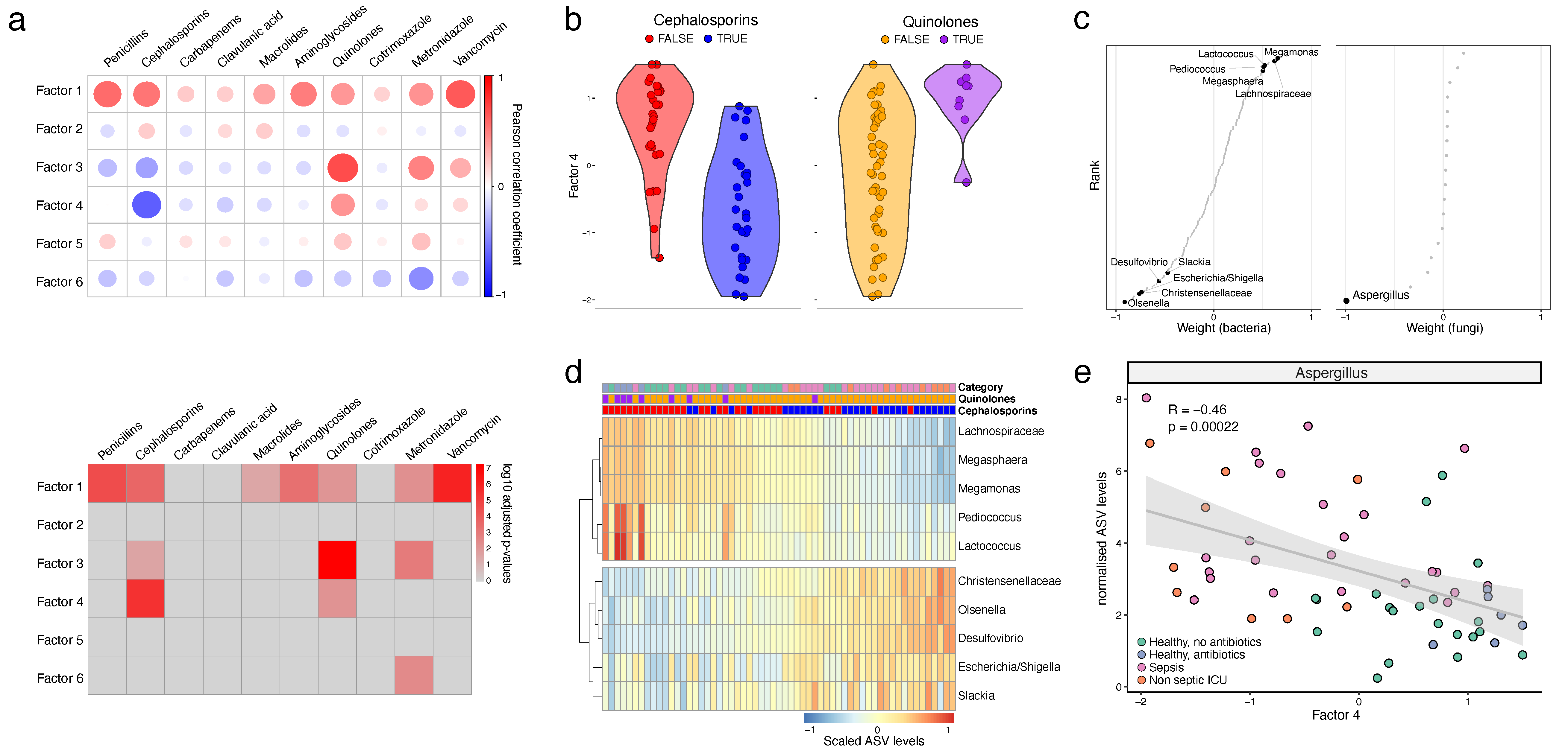

Supplement: FIG S6 [file msystems.01148-20-sf006.tif]

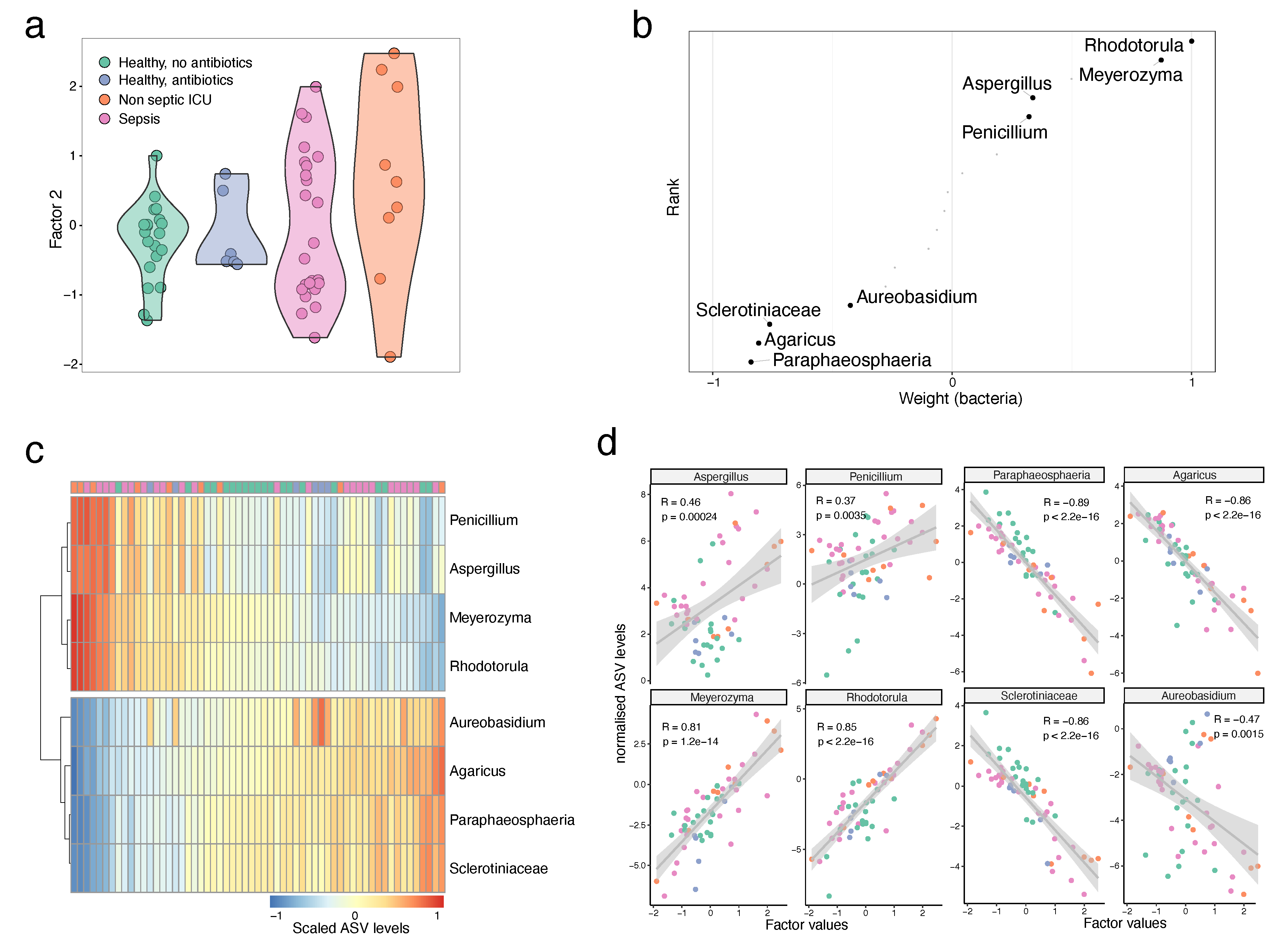

Supplement: FIG S7 [file msystems.01148-20-sf007.tif]
