# Supplementary material for: Integrative Transkingdom Analysis of the Gut Microbiome in Antibiotic Perturbation and Critical Illness
Source: mSystems. 2021 Mar 16;6(2):e01148-20. doi: 10.1128/mSystems.01148-20 (PMC8546997; doi:10.1128/mSystems.01148-20)
Supplement: TABLE S1 [file msystems.01148-20-st001.docx]

**Table S1. Characteristics of all included patients admitted to the ICU with sepsis or non-septic critical illness**

| **Age/ gender** | **Main diagnosis** | **APACHE IV*** | **SOFA*** | **Shock*** | **Vasoactive medication**** | **Renal failure**** | **Mechanical ventilation**** | **Acute Kidney Injury**** | **Acute Lung Injury**** | **In-hospital survival** | **90 day survival** |
| --- | --- | --- | --- | --- | --- | --- | --- | --- | --- | --- | --- |
| 89/ F | Sepsis, pulmonary | 99 | 10 | yes | yes | no | yes | no | no | Deceased | Deceased |
| 74/ F | Sepsis, pulmonary | 67 | 3 | no | no | no | yes | no | no | Alive | Deceased |
| 61/ M | Sepsis, pulmonary | 41 | 5 | no | no | no | yes | no | no | Alive | Alive |
| 67/ F | Sepsis, pulmonary | 40 | 6 | no | yes | no | yes | no | no | Alive | Alive |
| 72/ M | Sepsis, pulmonary | 90 | 6 | no | no | no | yes | yes | no | Deceased | Deceased |
| 57/ F | Sepsis, pulmonary | 24 | 3 | no | no | no | yes | no | no | Alive | Alive |
| 68/ M | Sepsis, pulmonary | 41 | 5 | no | no | no | yes | no | no | Alive | Alive |
| 62/ F | Sepsis, pulmonary | 107 | NA | yes | yes | no | yes | no | no | Alive | Alive |
| 71/ M | Sepsis, pulmonary | 79 | NA | no | yes | no | yes | no | no | Deceased | Deceased |
| 74/ M | Sepsis, pulmonary | 92 | 8 | yes | yes | no | yes | yes | yes | Alive | Alive |
| 66/ F | Sepsis, abdominal | 95 | 9 | yes | yes | no | no | yes | no | Deceased | Deceased |
| 69/ F | Sepsis, abdominal | 135 | 13 | yes | yes | no | yes | no | no | Deceased | Deceased |
| 59/ F | Sepsis, abdominal | 126 | 11 | yes | yes | no | yes | no | no | Alive | Alive |
| 46/ M | Sepsis, abdominal | 122 | 16 | yes | yes | no | yes | no | no | Deceased | Deceased |
| 64/ F | Sepsis, abdominal | 91 | 4 | no | yes | no | no | no | yes | Deceased | Deceased |
| 57/ M | Sepsis, abdominal | 81 | 9 | yes | yes | no | yes | yes | no | Alive | Alive |
| 70/ M | Sepsis, abdominal | 71 | 12 | yes | yes | yes | yes | no | no | Deceased | Deceased |
| 54/ F | Sepsis, urinary | 121 | 10 | no | yes | no | yes | no | no | Alive | Alive |
| 53/ F | Sepsis, urinary | 59 | 8 | yes | yes | no | no | no | no | Deceased | Deceased |
| 79/ M | Sepsis, urinary | 61 | NA | yes | yes | no | no | no | no | Deceased | Deceased |
| 50/ F | Sepsis, brain abscess | 91 | 5 | no | no | no | no | no | no | Alive | Deceased |
| 44/ M | Sepsis, meningitis | 59 | 10 | no | yes | yes | yes | no | no | Alive | Alive |
| 73/ M | Sepsis, mediastinitis | 88 | 8 | yes | yes | no | no | no | no | Alive | Alive |
| 79/ F | Sepsis, skin | 64 | 8 | no | yes | no | yes | no | no | Alive | Alive |
| 66/ M | Acute myocardial infarction | 77 | 9 | no | yes | no | yes | no | no | Deceased | Deceased |
| 65/ F | Overdose, alcohol | 95 | 5 | no | no | no | yes | no | no | Alive | Alive |
| 64/ F | Cardiogenic shock | 135 | 12 | yes | yes | yes | yes | no | no | Alive | Alive |
| 76/ M | Cardiac arrest | 91 | 5 | no | yes | no | yes | no | no | Alive | Alive |
| 79/ M | Dissection of aortic aneurysm | 116 | 10 | yes | yes | no | yes | no | no | Alive | Alive |
| 79/ F | Cardiac arrest | 102 | 8 | yes | yes | no | yes | no | yes | Alive | Deceased |
| 50/ F | Cardiac arrest | 97 | 8 | yes | yes | no | yes | no | no | Alive | Alive |
| 48/ F | Intracranial hemorrhage | 67 | 7 | no | yes | no | yes | no | no | Alive | Alive |
| 34/ M | Coma | 95 | 5 | no | no | no | yes | no | no | Alive | Alive |

* On admission; ** Within 24 hours of admission; NA, no data available
