# Supplementary material for: Integrative Transkingdom Analysis of the Gut Microbiome in Antibiotic Perturbation and Critical Illness
Source: mSystems. 2021 Mar 16;6(2):e01148-20. doi: 10.1128/mSystems.01148-20 (PMC8546997; doi:10.1128/mSystems.01148-20)
Supplement: TABLE S2 [file msystems.01148-20-st002.docx]

| **Protozoa** | **Sepsis**  **(n= 24)** | **Non-septic ICU (n=9)** | **Healthy, no antibiotics***  **(n =13)** | **Healthy, antibiotics^$^**  **(n =6)** |
| --- | --- | --- | --- | --- |
| *Giardia lambia*, n (%) | 1 (3.3%) | 0 (0) | 0 (0) | 0 (0) |
| *Crytosporidium parvum*, n (%) | 0 (0) | 0 (0) | 0 (0) | 0 (0) |
| *Entamoeba histolytica*, n (%) | 0 (0) | 0 (0) | 0 (0) | 0 (0) |
| *Blastocystis hominis*, n (%) | 3(12.5%) | 3 (33.3%) | 4 (30.7%) | 0 (0) |
| *Dientamoeba fragilis*, n (%) | 1 (4.2%) | 0 (0) | 4 (30.7%) | 0 (0) |

**Table S2. Overview of the intestinal presence of gut protozoa in all included patients**

*based on positive RT-PCR on sample collected on day 0 alone.

^$^based on positive RT-PCR on sample collected one day following 7-day course of oral antibiotics (day 9).
